# Selection of patients with ovarian cancer who may show survival benefit from hyperthermic intraperitoneal chemotherapy: A systematic review and meta-analysis

**DOI:** 10.1097/MD.0000000000018355

**Published:** 2019-12-16

**Authors:** Se Ik Kim, Jaehyun Cho, Eun Ji Lee, Sunwoo Park, Soo Jin Park, Aeran Seol, Nara Lee, Ga Won Yim, Maria Lee, Whasun Lim, Gwonhwa Song, Suk Joon Chang, Jae Won Kim, Hee Seung Kim

**Affiliations:** a Department of Obstetrics and Gynecology, Seoul National University College of Medicine; b Department of Obstetrics and Gynecology, Soon Chun Hyang University Hospital Seoul; c Institute of Animal Molecular Biotechnology and Department of Biotechnology, College of Life Sciences and Biotechnology, Korea University; d Department of Obstetrics and Gynecology, CHA Gangnam Medical Center, CHA University School of Medicine, Seoul; e Department of Food and Nutrition, Kookmin University, Seoul; f Gynecologic Cancer Center, Department of Obstetrics and Gynecology, Ajou University School of Medicine, Suwon, Republic of Korea.

**Keywords:** hyperthermic intraperitoneal chemotherapy, meta-analysis, ovarian cancer

## Abstract

**Background:**

The use of hyperthermic intraperitoneal chemotherapy (HIPEC) after cytoreductive surgery has been extensively studied in patients with peritoneal carcinomatosis from various malignancies. However, the effectiveness of HIPEC for ovarian cancer is still controversial. Therefore, we performed this meta-analysis to identify patients with ovarian cancer who can obtain survival benefit from HIPEC.

**Methods:**

Articles regarding HIPEC in the MEDLINE, EMBASE, and Cochrane Library were searched till December 2018. In total, 13 case-control studies and two randomized controlled trials were included in this meta-analysis. We investigated the effect of HIPEC on disease-free survival (DFS) and overall survival (OS), and performed subgroup analyses based on the study design, adjustment of confounding variables, and quality of the study.

**Results:**

HIPEC improved both DFS (hazard ratio [HR], 0.603; 95% confidence interval [CI], 0.513–0.709) and OS (HR, 0.640; 95% CI, 0.519–0.789). In cases of primary disease, HIPEC improved DFS (HR, 0.580; 95% CI, 0.476–0.706) and OS (HR, 0.611; 95% CI, 0.376–0.992). Subgroup analyses revealed that HIPEC did not improve OS but improved DFS of patients with residual tumors ≤1 cm or no visible tumors. In cases of recurrent disease, HIPEC was associated with better OS (HR, 0.566; 95% CI, 0.379–0.844) but not with DFS. Subgroup analyses also revealed similar tendencies. However, HIPEC improved DFS of patients with residual tumors ≤1 cm or no visible tumors, while it improved OS of only those with residual tumors ≤1 cm.

**Conclusions:**

HIPEC may improve DFS of patients with ovarian cancer when residual tumors were ≤1 cm or not visible. It may also improve OS of only patients with recurrent disease whose residual tumors were ≤1 cm.

## Abstract 2

Supplemental Digital Content is available in the text

## 1 Introduction

Peritoneal carcinomatosis develops in more than 80% of patients with advanced ovarian cancer, resulting in a 5-year survival rate of <50%.^[1,2]^ In terms of the biologic aspect of intraperitoneal dissemination of tumors, peritoneal carcinomatosis is considered the terminal status of cancers, resulting in poor prognosis. However, there is no effective method for treating peritoneal carcinomatosis from most solid tumors, and both surgical resection and systemic chemotherapy have shown minimal effects on survival.^[3,4]^

In particular, hyperthermic intraperitoneal chemotherapy (HIPEC) after cytoreductive surgery has been extensively studied in patients with peritoneal carcinomatosis from various malignancies, with an improvement in the survival rate and reduction in the recurrence rate.^[5,6]^ Compared to conventional intraperitoneal chemotherapy, HIPEC has several advantages, even showing synergistic effects. Hyperthermia itself has direct cytotoxicity on tumors and increases the penetration of chemotherapy and drug concentration at the peritoneal surface. Moreover, HIPEC can decrease catheter-related complications observed after conventional intraperitoneal chemotherapy because it is conducted in a single session.^[7]^

Till date, only 2 randomized controlled trials (RCTs) have evaluated the effect and safety of HIPEC for ovarian cancer.^[8,9]^ Spiliotis et al reported that HIPEC resulted in survival benefit for patients with recurrent ovarian cancer.^[8]^ However, that study had limitations considering the randomization process and the definition of the end-points, both of which affect the interpretation of the results.^[10]^ In the other RCT performed by van Driel et al, better disease-free survival (DFS) and overall survival (OS) were observed in patients treated with neoadjuvant chemotherapy (NAC) followed by interval debulking surgery (IDS) and HIPEC, compared to those treated with NAC followed by IDS alone.^[9]^ However, the small sample size resulted in an inter-group difference of only 15 deaths, and the different effects of HIPEC among centers make it hard to justify the practical application of HIPEC in the clinical setting.^[11]^ Moreover, a previous meta-analysis did not provide the exact pooled hazard ratios (HRs) associated with HIPEC for evaluating the effect.^[12]^

Thus, precise knowledge regarding the exact impact of HIPEC on the prognosis of ovarian cancer is still needed, owing to the heterogeneity in the study population, such as primary or recurrent disease, and the extent of cytoreductive surgery among the previous studies. In particular, the identification of patients with ovarian cancer who can benefit from HIPEC will allow for the implementation of individualized treatment. For this purpose, we performed a meta-analysis to investigate the effect of HIPEC on the survival of patients with ovarian cancer.

## 2 Methods

### 2.1 Search strategy and selection criteria

This meta-analysis was conducted in accordance with the recommendations per the Preferred Reporting Items for Systematic Reviews and Meta-Analyses guidelines.^[13]^ Studies investigating the effect of HIPEC on the prognosis of ovarian cancer were identified via a literature search of the MEDLINE, EMBASE, and Cochrane Library, from when recording began up to December 2018. Our overall search strategy included the following terms for HIPEC (“hyperthermic intraperitoneal chemotherapy” or “HIPEC” or “intraperitoneal”), ovary (“ovarian” or “ovary”), and cancer (“cancer” or “carcinoma” or “neoplasm” or “malignancy”, or “tumor”). Details about the search strategy are shown in Supplementary Table 1.

We included relevant studies that met the following criteria: studies that included patients with epithelial ovarian cancer; study designs included RCT, case-control, and 2-arm cohort studies; and comparison of DFS or OS between patients who underwent HIPEC and those who did not receive it. However, we excluded the following studies: review articles, case reports, editorials, and letters to the editor; studies that had no data of survival or did not meet the selection criteria; and non-English literature.

As the present meta-analysis was performed based on previously published studies, thus no ethical approval and patient consent are required.

### 2.2 Selection of studies

**Figure 1:**
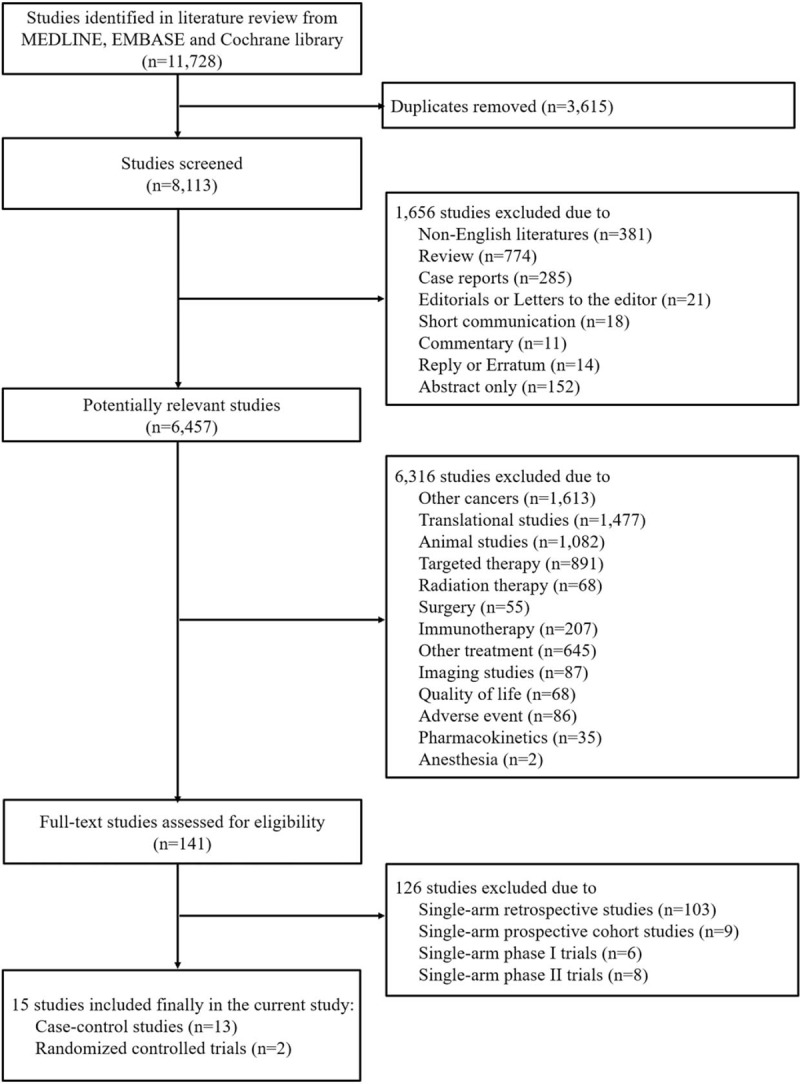
The search strategy and number of studies identified for inclusion in this meta-analysis.

Two authors (SIK and SJP) independently screened the eligibility of all studies retrieved from the database according to the predetermined selection criteria. The third author (HSK) resolved any disagreement between the 2 authors after discussion. A total of 11,728 studies were identified, and we excluded 3615 duplicates. We excluded 7972 studies because of the following reasons: non-English literature (n = 381), non-original articles (n = 1275), studies on other cancers (n = 1613), translational studies (n = 1477), animal studies (n = 1082), studies on other treatment modalities (n = 1866), and studies dealing with other issues (n = 278). In addition, we excluded 126 non-relevant articles after assessing the full-text articles. Finally, 13 case-control studies^[14–26]^ and 2 RCTs^[8,9]^ with 1314 patients were included in the meta-analysis (Fig. 1).

### 2.3 Data extraction

Two authors (SIK and EJL) independently extracted the data, and any discrepancies were addressed by a joint re-evaluation of the article with the third author (HSK). The following data were extracted from each study for the meta-analysis: author; year of publication; country in which the study was performed; study design; disease status (primary disease, platinum-sensitive and platinum-resistant recurrence); the International Federation of Gynecology and Obstetrics (FIGO) stage; histology; grade; age; numbers of patients who received HIPEC and who did not receive it; drugs and methods of HIPEC; the extent of cytoreductive surgery (or residual tumor size after cytoreductive surgery); follow-up period; DFS and OS; and HRs with 95% confidence intervals (CIs).

For the study with only the HR and *P* value of the Cox proportional hazards model,^[14]^ we estimated the 95% CI mathematically. If patients treated with HIPEC were regarded as the reference group, the HRs were inverted and 95% CIs were subsequently calculated.^[16,22,26]^ In case of studies in which the risk parameters were not presented with specific numbers, we could obtain the estimated risks with 95% CIs by analyzing survival curves^[8,15,18–20,24–26]^ according to the statistical procedure described by Tierney et al.^[27]^

### 2.4 Quality assessment

The methodological quality of the 13 case-control studies were evaluated based on the Newcastle-Ottawa Scale (NOS).^[28]^ The NOS includes eight items over three dimensions: selection, comparability, and exposure with a maximum of 4, 2, and 3 points, respectively. In this meta-analysis, 11 of 13 case-control studies scored 8 showing “high quality”, whereas the other 2 studies scored 6 showing “low quality” (Supplementary Table 2).

### 2.5 Statistical analysis

Pooled HRs with 95% CIs were calculated in all studies, and heterogeneity was assessed by using the Higgins *I*^*2*^ value that represented the percentage of the total variance in the summary estimate owing to inter-study heterogeneity rather than chance.^[29]^ A value of > 50% was considered to have substantial heterogeneity, and we used the random effects model with the DerSimonian and Laird method. When the *I*^*2*^ value was ≤50%, we used the fixed effect model with the Mantel–Haenszel method. In the fixed effect model, each study was weighted by the inverse of its variance.

Subgroup meta-analyses were performed based on the study design, adjustment of confounding variables, and quality of the study. To identify the publication bias, funnel plots were used, where each study's HR and standard error of the log HR were plotted on the X-axis and Y-axis, respectively. We observed symmetric funnel plots, implying no publication bias in this meta-analysis. The Egger test results also showed the absence of publication bias (Supplementary Figure 1).

All statistical analyses were performed with Comprehensive Meta-analysis Version 2.0 (Biostat Inc., Englewood, NJ), and a *P* < .05 was considered statistically significant. All statistical tests were two-sided.

## 3 Results

### 3.1 Effect of HIPEC on survival by study design

**Figure 2:**
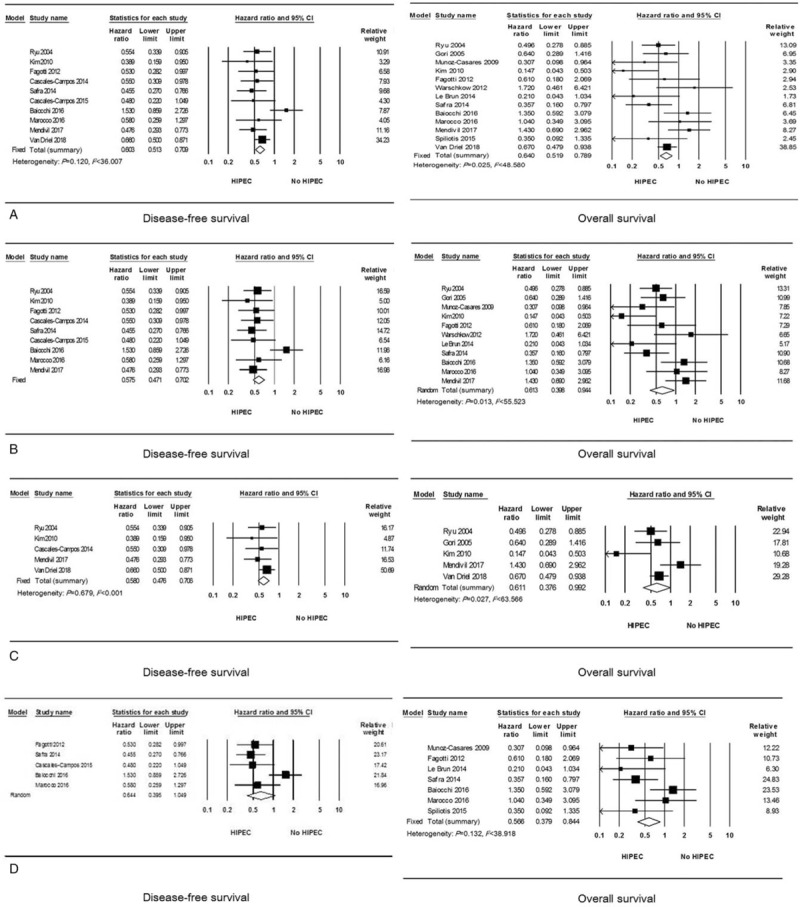
Effect of hyperthermic intraperitoneal chemotherapy (HIPEC) on survival by study design: (A) all studies; (B) case-control studies, and by disease status: (C) primary disease; (D) recurrent disease.

The characteristics of the 13 case-control studies and two RCTs including 1,314 patients are shown in Table 1. Potential confounding variables such as age, FIGO stage, histology, grade, and residual tumor size at the first surgery were adjusted in most of the studies. In all the studies, HIPEC improved both DFS (HR, 0.603; 95% CI, 0.513–0.709) and OS (HR, 0.640; 95% CI, 0.519–0.789; Fig. 2A). On subgroup analyses confined to the case-control studies, HIPEC improved DFS (HR, 0.575; 95% CI, 0.471–0.702)^[14,17,18,20,22–26]^ and OS (HR, 0.613; 95% CI, 0.398–0.944; Fig. 2B).^[14–19,21,22,24–26]^

**Table 1:**
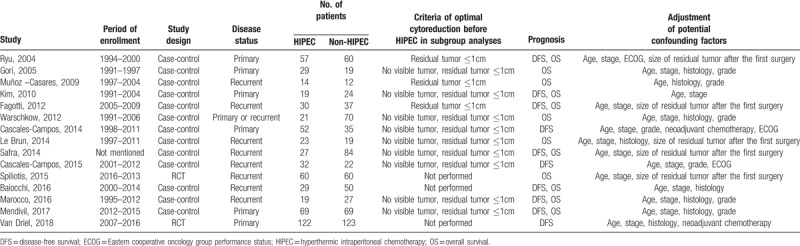
Characteristics of 15 studies about the effect of hyperthermic intraperitoneal chemotherapy on prognosis of ovarian cancer.

### 3.2 Effect of HIPEC on survival by disease status

For cases of primary disease, five studies including 630 patients showed that HIPEC was associated with better DFS (HR, 0.580; 95% CI, 0.476–0.706),^[9,14,17,20,26]^ and 5 studies including 591 patients also showed that HIPECT was associated with improved OS (HR, 0.611; 95% CI, 0.376–0.992; Fig. 2C).^[9,14,15,17,26]^ When we performed subgroup analyses according to the study design, FIGO stage, and adjustment of confounding variables, HIPEC showed a favorable effect on DFS, whereas it failed to improve OS. However, HIPEC showed a favorable effect on OS for advanced, stage III-IV disease (HR, 0.748; 95% CI, 0.563–0.994; Table 2).^[9,15,26]^

**Table 2:**
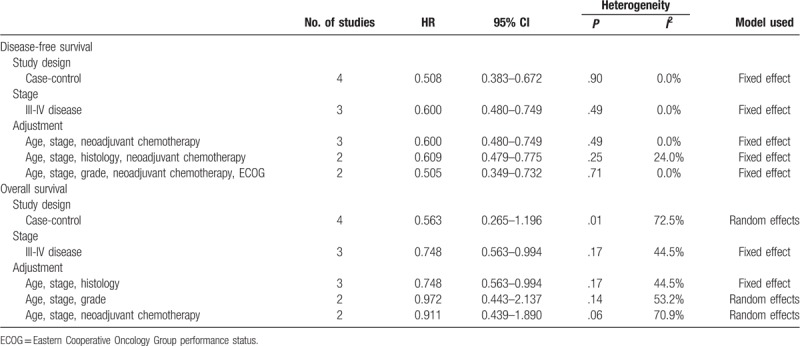
Subgroup analyses for evaluating the effect of hyperthermic intraperitoneal chemotherapy on the survival of patients with primary disease.

For cases of recurrent disease, 5 studies including 357 patients did not show improved DFS after HIPEC (HR, 0.644; 95% CI, 0.395–1.049).^[18,22–25]^ ln particular, all these 5 studies targeted platinum-sensitive recurrent disease. On subgroup analyses according to the study design and quality of study, HIPEC failed to improve DFS. However, HIPEC showed better DFS after adjusting confounding variables (Table 3).

**Table 3:**
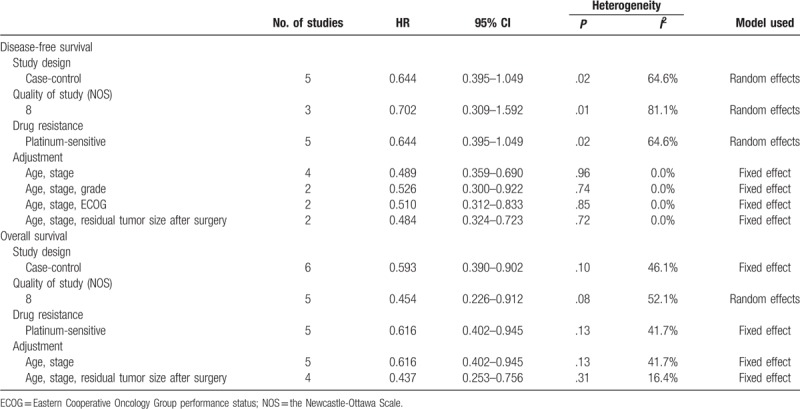
Subgroup analyses for evaluating the effect of hyperthermic intraperitoneal chemotherapy on the survival of patients with recurrent disease.

In terms of OS of patients with recurrent disease, 7 studies including 491 patients showed survival benefit after HIPEC (HR, 0.566; 95% CI, 0.379–0.844; Fig. 2D).^[8,16,18,21,22,24,25]^ When meta-analysis was performed by including only 5 studies that targeted platinum-sensitive recurrent disease, HIPEC also showed a favorable effect on OS (HR, 0.616; 95% CI, 0.402–0.945).^[18,21,22,24,25]^ On subgroup analyses according to the study design, quality of study, and adjustment of confounding variables, HIPEC was consistently associated with better OS (Table 3).

### 3.3 Effect of HIPEC on survival by the extent of cytoreductive surgery

HIPEC significantly prolonged the DFS of patients with residual tumors ≤1 cm after cytoreductive surgery (HR, 0.488; 95% CI, 0.389–0.612)^[14,17,18,20,22,23,25,26]^ and in those with no visible tumor (HR, 0.486; 95% CI, 0.377–0.628).^[17,20,22,23,25,26]^ These results were also observed on subgroup analyses according to disease status, quality of the study, and adjustment of confounding variables (Table 4).

**Table 4:**
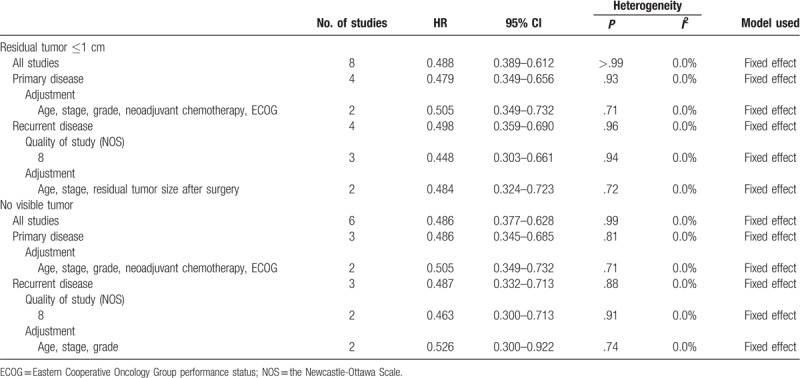
Effect of hyperthermic intraperitoneal chemotherapy on disease-free survival by the extent of cytoreductive surgery.

However, HIPEC did not increase OS of patients with no visible tumor (HR, 0.564; 95% CI, 0.310–1.027)^[15,17,19,21,22,25,26]^ despite the improvement of OS of those with residual tumors ≤1 cm after cytoreductive surgery (HR, 0.591; 95% CI, 0.431–0.811).^[14–19,21,22,25,26]^ On subgroup analyses, HIPEC was effective for patients with recurrent disease who had residual tumors ≤1 cm after cytoreductive surgery (HR, 0.493; 95% CI, 0.315–0.773; Table 5).^[16,18,19,21,22,25]^

**Table 5:**
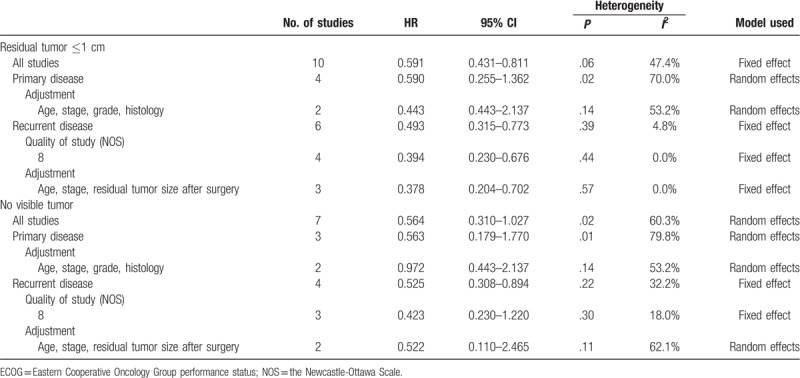
Effect of hyperthermic intraperitoneal chemotherapy on overall survival by the extent of cytoreductive surgery.

## 4 Discussion

The current meta-analysis provides further evidence that HIPEC may be associated with better survival of patients with ovarian cancer, and suggests how we can select patients with ovarian cancer who will benefit from HIPEC after cytoreductive surgery.

Considering the DFS, HIPEC was associated with better prognosis in patients with primary disease, whereas it failed to increase DFS of patients with recurrent disease. However, subgroup analyses revealed that HIPEC increased DFS of patients with residual tumors ≤1 cm and no visible tumor, regardless of primary or recurrent diseases. These results suggest that HIPEC may be effective for all patients with primary ovarian cancer, whereas its effect may be limited for those who underwent optimal cytoreduction (residual tumors ≤1 cm and no visible tumor) for recurrent disease. The survival benefit from HIPEC in primary disease is in line with the RCT of van Driel et al in which HIPEC increased DFS of patients with ovarian cancer who received NAC followed by IDS.^[9]^ After NAC, hidden tumors might still exist despite gross evaluation and optimal cytoreduction after IDS.^[30]^ However, HIPEC may control both biologically residual and hidden tumors, resulting in a favorable prognosis.

For patients with recurrent ovarian cancer, improvement of DFS after HIPEC was observed only in those who achieved optimal cytoreductive surgery in this study. This limitation might have originated owing to the different biological properties of recurrent tumors because they commonly show drug resistance to chemotherapy.^[31]^ Moreover, the penetration depth of chemotherapeutic drugs in HIPEC is limited to a few millimeters only.^[32]^ Accordingly, the role of cytoreductive surgery may be particularly important for recurrent ovarian cancer, and optimal cytoreduction should be performed before the implementation of HIPEC because of drug resistance and limited penetration depth of the drugs used in HIPEC.

In terms of OS, HIPEC improved the prognosis in both primary and recurrent diseases. However, the effect of HIPEC was not observed in patients with primary disease who had residual tumors ≤1 cm or no visible tumors. In cases of primary disease, most of the tumors are naïve to systemic chemotherapy. In addition, we have to keep in mind that HIPEC has treatment-related complications as well.^[33]^ Therefore, HIPEC might be unnecessary for patients with primary disease if optimal cytoreductive surgery is achieved and completion of planned cycles of adjuvant chemotherapy is expected.

The current meta-analysis showed that HIPEC did not increase OS of patients with recurrent ovarian cancer who had no visible tumor after cytoreductive surgery. However, the effect of HIPEC on OS could be expected in those who had residual tumors ≤1 cm after cytoreductive surgery. We do not know the exact reason, but one it is possible that HIPEC can increase the response of drug-resistant tumor cells to systemic chemotherapy. Previous studies have suggested that drug-resistant tumor cells with high amount of heat-shock proteins became more susceptible to the effect of hyperthermia,^[34]^ and epigenetic alterations induced by hyperthermic chemo-perfusion altered the responsiveness to platinum agents.^[35]^

Nevertheless, this meta-analysis had some limitations. First, the different types of drugs used in HIPEC among the studies may result in bias. Second, the toxicity or adverse events of HIPEC were not evaluated. Third, most studies in this meta-analysis were retrospective studies except for the 2 RCTs.

Despite these limitations, the results of the current meta-analysis suggest the strong relationship between HIPEC and better survival of patients with primary or recurrent ovarian cancer. In particular, the results of this meta-analysis are significant, as they indicate which patients with ovarian cancer may benefit from cytoreductive surgery and HIPEC. However, additional relevant clinical trials are needed to select the appropriate patients and to demonstrate the effect of HIPEC on their prognosis in the near future.

## Author contributions

**Conceptualization:** Se Ik Kim, Whasun Lim, Hee Seung Kim.

**Data curation:** Se Ik Kim, Hee Seung Kim.

**Formal analysis:** Se Ik Kim, Eun Ji Lee, Hee Seung Kim.

**Funding acquisition:** Hee Seung Kim.

**Investigation:** Hee Seung Kim.

**Methodology:** Se Ik Kim, Eun Ji Lee, Soo Jin Park, Hee Seung Kim.

**Supervision:** Gwonhwa Song, Suk Joon Chang, Jae Won Kim, Hee Seung Kim.

**Validation:** Se Ik Kim, Eun Ji Lee, Soo Jin Park, Hee Seung Kim.

**Writing - Original Draft:** Se Ik Kim, Hee Seung Kim.

**Writing - Review & Editing:** Se Ik Kim, Jaehyun Cho, Sunwoo Park, Soo Jin Park, Aeran Seol, Nara Lee, Ga Won Yim, Maria Lee, Whasun Lim, Gwonhwa Song, Suk Joon Chang, Jae Won Kim, Hee Seung Kim.

Hee Seung Kim orcid: 0000-0001-6876-8671.

## Supplementary Material

Supplemental Digital Content

## Supplementary Material

Supplemental Digital Content

## Supplementary Material

Supplemental Digital Content

## References

[1] Siegel RL Miller KD Jemal A Cancer Statistics, 2017. CA Cancer J Clin 2017;67:7–30. 28055103 10.3322/caac.21387

[2] National Cancer Institute: SEER Cancer Statistics Review (CSR) 1975–2014. https://seer.cancer.gov/csr/1975_2014/, available at 1 Dec 2019.

[3] Coccolini F Gheza F Lotti M Peritoneal carcinomatosis. World J Gastroenterol 2013;19:6979–94. 24222942 10.3748/wjg.v19.i41.6979 PMC3819534

[4] Sugarbaker PH Carcinomatosis--is cure an option? J Clin Oncol 2003;21:762–4. 12610170 10.1200/JCO.2003.12.071

[5] Elias D Lefevre JH Chevalier J Complete cytoreductive surgery plus intraperitoneal chemohyperthermia with oxaliplatin for peritoneal carcinomatosis of colorectal origin. J Clin Oncol 2009;27:681–5. 19103728 10.1200/JCO.2008.19.7160

[6] Mi DH Li Z Yang KH Surgery combined with intraoperative hyperthermic intraperitoneal chemotherapy (IHIC) for gastric cancer: a systematic review and meta-analysis of randomised controlled trials. Int J Hyperthermia 2013;29:156–67. 23418917 10.3109/02656736.2013.768359

[7] Witkamp AJ de Bree E Van Goethem R Rationale and techniques of intra-operative hyperthermic intraperitoneal chemotherapy. Cancer Treat Rev 2001;27:365–74. 11908929 10.1053/ctrv.2001.0232

[8] Spiliotis J Halkia E Lianos E Cytoreductive surgery and HIPEC in recurrent epithelial ovarian cancer: a prospective randomized phase III study. Ann Surg Oncol 2015;22:1570–5. 25391263 10.1245/s10434-014-4157-9

[9] van Driel WJ Koole SN Sikorska K Hyperthermic intraperitoneal chemotherapy in ovarian cancer. N Engl J Med 2018;378:230–40. 29342393 10.1056/NEJMoa1708618

[10] Harter P Reuss A Sehouli J Brief report about the role of hyperthermic intraperitoneal chemotherapy in a prospective randomized phase 3 study in recurrent ovarian cancer from Spiliotis et al. Int J Gynecol Cancer 2017;27:246–7. 10.1097/IGC.0000000000000864 28114231

[11] Vergote I Chiva L du Bois A Hyperthemic intraperitoneal chemotherapy in ovarian cancer. N Engl J Med 2018;378:1362–3. 10.1056/NEJMc1802033 29619815

[12] Huo YR Richards A Liauw W Hyperthermic intraperitoneal chemotherapy (HIPEC) and cytoreductive surgery (CRS) in ovarian cancer: a systematic review and meta-analysis. Eur J Surg Oncol 2015;41:1578–89. 26453145 10.1016/j.ejso.2015.08.172

[13] Liberati A Altman DG Tetzlaff J The PRISMA statement for reporting systematic reviews and meta-analyses of studies that evaluate health care interventions: explanation and elaboration. J Clin Epidemiol 2009;62:e1–34. 19631507 10.1016/j.jclinepi.2009.06.006

[14] Ryu KS Kim JH Ko HS Effects of intraperitoneal hyperthermic chemotherapy in ovarian cancer. Gynecol Oncol 2004;94:325–32. 15297169 10.1016/j.ygyno.2004.05.044

[15] Gori J Castano R Toziano M Intraperitoneal hyperthermic chemotherapy in ovarian cancer. Int J Gynecol Cancer 2005;15:233–9. 10.1111/j.1525-1438.2005.15209.x 15823105

[16] Munoz-Casares FC Rufian S Rubio MJ The role of hyperthermic intraoperative intraperitoneal chemotherapy (HIPEC) in the treatment of peritoneal carcinomatosis in recurrent ovarian cancer. Clin Transl Oncol 2009;11:753–9. 19917539 10.1007/s12094-009-0438-3

[17] Kim JH Lee JM Ryu KS Consolidation hyperthermic intraperitoneal chemotherapy using paclitaxel in patients with epithelial ovarian cancer. J Surg Oncol 2010;101:149–55. 20035540 10.1002/jso.21448

[18] Fagotti A Costantini B Petrillo M Cytoreductive surgery plus HIPEC in platinum-sensitive recurrent ovarian cancer patients: a case-control study on survival in patients with two year follow-up. Gynecol Oncol 2012;127:502–5. 23022234 10.1016/j.ygyno.2012.09.020

[19] Warschkow R Tarantino I Lange J Does hyperthermic intraoperative chemotherapy lead to improved outcomes in patients with ovarian cancer? A single center cohort study in 111 consecutive patients. Patient Saf Surg 2012;6:12. 22709648 10.1186/1754-9493-6-12 PMC3407737

[20] Cascales-Campos PA Gil J Gil E Treatment of microscopic disease with hyperthermic intraoperative intraperitoneal chemotherapy after complete cytoreduction improves disease-free survival in patients with stage IIIC/IV ovarian cancer. Ann Surg Oncol 2014;21:2383–9. 24599409 10.1245/s10434-014-3599-4

[21] Le Brun JF Campion L Berton-Rigaud D Survival benefit of hyperthermic intraperitoneal chemotherapy for recurrent ovarian cancer: a multi-institutional case control study. Ann Surg Oncol 2014;21:3621–7. 24819120 10.1245/s10434-014-3693-7

[22] Safra T Grisaru D Inbar M Cytoreduction surgery with hyperthermic intraperitoneal chemotherapy in recurrent ovarian cancer improves progression-free survival, especially in BRCA-positive patients- a case-control study. J Surg Oncol 2014;110:661–5. 24962381 10.1002/jso.23688

[23] Cascales-Campos PA Gil J Feliciangeli E The role of hyperthermic intraperitoneal chemotherapy using paclitaxel in platinum-sensitive recurrent epithelial ovarian cancer patients with microscopic residual disease after cytoreduction. Ann Surg Oncol 2015;22:987–93. 25212832 10.1245/s10434-014-4049-z

[24] Baiocchi G Ferreira FO Mantoan H Hyperthermic intraperitoneal chemotherapy after secondary cytoreduction in epithelial ovarian cancer: a single-center comparative analysis. Ann Surg Oncol 2016;23:1294–301. 26628430 10.1245/s10434-015-4991-4

[25] Marocco F Vaira M Milani A Secondary cytoreductive surgery, hyperthermic intraperitoneal intraoperative chemotherapy, and chemotherapy alone: a retrospective comparison of alternative approaches in relapsed platinum sensitive ovarian cancer. Eur J Gynaecol Oncol 2016;37:638–43. 29787001

[26] Mendivil AA Rettenmaier MA Abaid LN Consolidation hyperthermic intraperitoneal chemotherapy for the treatment of advanced stage ovarian carcinoma: a 3 year experience. Cancer Chemother Pharmacol 2017;80:405–10. 28669065 10.1007/s00280-017-3376-8

[27] Tierney JF Stewart LA Ghersi D Practical methods for incorporating summary time-to-event data into meta-analysis. Trials 2007;8:16. 17555582 10.1186/1745-6215-8-16 PMC1920534

[28] Wells GA SB, O’Connell D, Peterson J, et al. The Newcastle-Ottawa Scale (NOS) for assessing the quality of nonrandomised studies in meta-analyses. http://www.ohri.ca/programs/clinical_epidemiology/oxford.asp, available at 1 Dec 2019.

[29] Higgins JP Thompson SG Deeks JJ Measuring inconsistency in meta-analyses. BMJ 2003;327:557–60. 12958120 10.1136/bmj.327.7414.557 PMC192859

[30] Markauskas A Mogensen O dePont Christensen R Primary surgery or interval debulking for advanced epithelial ovarian cancer: does it matter? Int J Gynecol Cancer 2014;24:1420–8. 25180461 10.1097/IGC.0000000000000241

[31] Vasey PA Resistance to chemotherapy in advanced ovarian cancer: mechanisms and current strategies. Br J Cancer 2003;89: Suppl 3: S23–28. 14661043 10.1038/sj.bjc.6601497 PMC2750620

[32] van de Vaart PJ van der Vange N Zoetmulder FA Intraperitoneal cisplatin with regional hyperthermia in advanced ovarian cancer: pharmacokinetics and cisplatin-DNA adduct formation in patients and ovarian cancer cell lines. Eur J Cancer 1998;34:148–54. 9624250 10.1016/s0959-8049(97)00370-5

[33] Zivanovic O Chi DS Filippova O It's time to warm up to hyperthermic intraperitoneal chemotherapy for patients with ovarian cancer. Gynecol Oncol 2018;151:555–61. 30249527 10.1016/j.ygyno.2018.09.007 PMC6684262

[34] Didelot C Lanneau D Brunet M Anti-cancer therapeutic approaches based on intracellular and extracellular heat shock proteins. Curr Med Chem 2007;14:2839–47. 18045130 10.2174/092986707782360079

[35] Hegyi G Szigeti GP Szasz A Hyperthermia versus oncothermia: cellular effects in complementary cancer therapy. Evid Based Complement Altern Med 2013;2013:672873. 10.1155/2013/672873 PMC3638606 23662149

