# Supplementary material for: Selection of patients with ovarian cancer who may show survival benefit from hyperthermic intraperitoneal chemotherapy: A systematic review and meta-analysis
Source: Medicine (Baltimore). 2019 Dec 16;98(50):e18355. doi: 10.1097/MD.0000000000018355 (PMC6922570; doi:10.1097/MD.0000000000018355)
Supplement: Supplemental Digital Content [file medi-98-e18355-s001.docx]

**Supplementary Table 1.** Search strategy

**Search strategy for MEDLINE**

#1. “hyperthermic intraperitoneal chemotherapy” [All fields]

#2. “HIPEC” [All fields]

#3. “intraperitoneal” [All fields]

#4. ((#1) OR #2) OR #3

#5. “ovarian” [All fields]

#6. “ovary” [All fields]

#7. (#5) OR #6

#8. “cancer” [All fields]

#9. “carcinoma” [All fields]

#10. “neoplasm” [All fields]

#11. “malignancy” [All fields]

#12. “tumor” [All fields]

#13. ((((#8) OR #9) OR #10) OR #11) OR #12

#14. (((#4) AND #7) AND #13)

Date of Search: December 31, 2018

Result: 3,616 articles found

**Search strategy for EMBASE**

#1. ‘hyperthermic intraperitoneal chemotherapy’/exp

#2. ‘HIPEC’/exp

#3. ‘intraperitoneal’/exp

#4. #1 OR #2 OR #3

#5. ‘ovarian’/exp

#6. ‘ovary’/exp

#7. #5 OR #6

#8. ‘cancer’/exp

#9. ‘carcinoma’/exp

#10. ‘neoplasm’/exp

#11. ‘malignancy’/exp

#12. ‘tumor’/exp

#13. #8 OR #9 OR #10 OR #11 OR #12

#14. #4 AND #7 AND #13

Date of Search: December 31, 2018

Result: 7,796 articles found

**Search strategy for the Cochrane Library**

#1. “hyperthermic intraperitoneal chemotherapy”

#2. “HIPEC”

#3. “intraperitoneal”

#4. #1 or #2 or #3

#5. “ovarian”

#6. “ovary”

#7. #5 or #6

#8. “cancer”

#9. “carcinoma”

#10. “neoplasm”

#11. “malignancy”

#12. “tumor”

#13. #8 or #9 or #10 or #11 or #12

#14. #4 and #7 and #13

Limitations: Title, Abstract, Keywords

Date of Search: December 31, 2018

Result: 316 articles found
