# Supplementary material for: Selection of patients with ovarian cancer who may show survival benefit from hyperthermic intraperitoneal chemotherapy: A systematic review and meta-analysis
Source: Medicine (Baltimore). 2019 Dec 16;98(50):e18355. doi: 10.1097/MD.0000000000018355 (PMC6922570; doi:10.1097/MD.0000000000018355)
Supplement: Supplemental Digital Content [file medi-98-e18355-s002.docx]

**Supplementary Table 2.** The Newcastle-Ottawa Scale (NOS) for assessing the qualities of the 13 case-control studies

| Study | Selection  (score) | | | | Comparability  (score) | Exposure  (score) | | | Total score |
| --- | --- | --- | --- | --- | --- | --- | --- | --- | --- |
|  | Adequate definition of cases | Representativeness of cases | Selection of control | Definition of controls | Control for important or additional factor | Ascertainment of exposure | Same method of ascertainment for cases and controls | Non-response rate |  |
| Ryu 2004 | 1 | 1 | 1 | 1 | 2 | 1 | 1 | 0 | 8 |
| Gori 2005 | 1 | 1 | 1 | 1 | 2 | 1 | 1 | 0 | 8 |
| Muñoz-Casares 2009 | 1 | 1 | 1 | 1 | 2 | 1 | 1 | 0 | 8 |
| Kim 2010 | 1 | 1 | 1 | 1 | 2 | 1 | 1 | 0 | 8 |
| Fagotti 2012 | 1 | 1 | 1 | 1 | 0 | 1 | 1 | 0 | 6 |
| Warschkow 2012 | 1 | 1 | 1 | 1 | 2 | 1 | 1 | 0 | 8 |
| Cascales-Campos 2014 | 1 | 1 | 1 | 1 | 2 | 1 | 1 | 0 | 8 |
| Le Brun 2014 | 1 | 1 | 1 | 1 | 2 | 1 | 1 | 0 | 8 |
| Safra 2014 | 1 | 1 | 1 | 1 | 2 | 1 | 1 | 0 | 8 |
| Cascales-Campos 2015 | 1 | 1 | 1 | 1 | 2 | 1 | 1 | 0 | 8 |
| Baiocchi 2016 | 1 | 1 | 1 | 1 | 2 | 1 | 1 | 0 | 8 |
| Marocco 2016 | 1 | 1 | 1 | 1 | 0 | 1 | 1 | 0 | 6 |
| Mendivil 2017 | 1 | 1 | 1 | 1 | 2 | 1 | 1 | 0 | 8 |
