# Supplementary material for: Selection of patients with ovarian cancer who may show survival benefit from hyperthermic intraperitoneal chemotherapy: A systematic review and meta-analysis
Source: Medicine (Baltimore). 2019 Dec 16;98(50):e18355. doi: 10.1097/MD.0000000000018355 (PMC6922570; doi:10.1097/MD.0000000000018355)

**Supplementary Figure 1.** Assessment of publication bias in this meta-analysis. (A-D) Impact of HIPEC on disease-free survival in ovarian cancer: (A) all studies; (B) case-control studies; (C) primary disease; (D) recurrent disease. (E-H) Impact of HIPEC on overall survival in ovarian cancer: (E) all studies; (F) case-control studies; (G) primary disease; (H) recurrent disease.


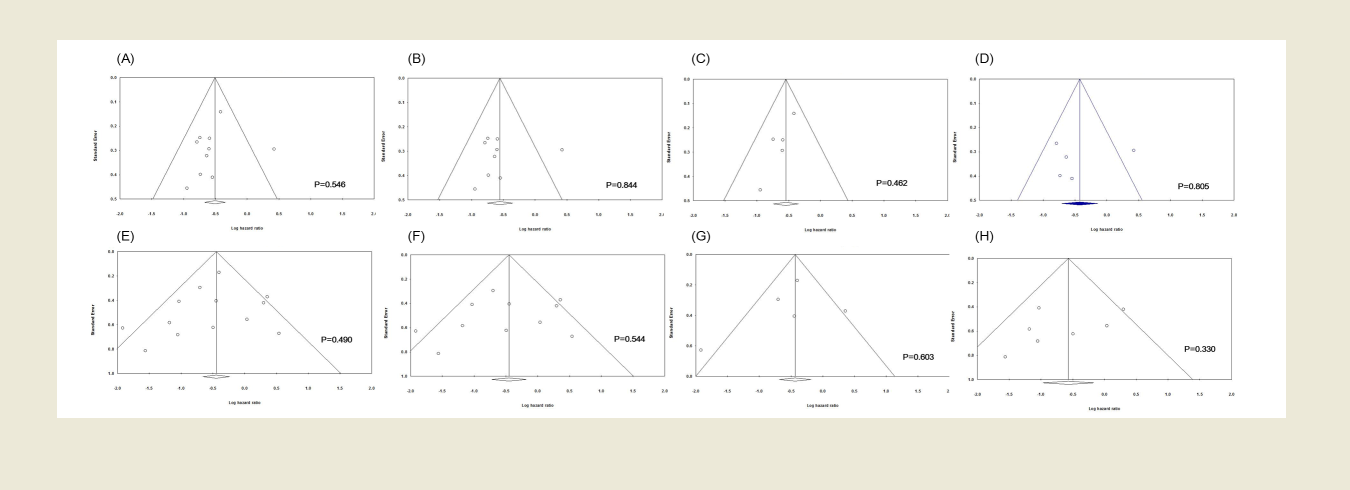

Supplement: Supplemental Digital Content [file medi-98-e18355-s003.doc]
